# An unusual path to diagnosis: Sertoli cell neoplasia revealing Peutz-Jeghers syndrome in an 8-year-old boy

**DOI:** 10.1016/j.eucr.2025.103211

**Published:** 2025-09-15

**Authors:** Rashed Almusalam, Shaikha Janahi, Mohammed Basem, Hasan Isa, Abdulrahman Alshafei

**Affiliations:** a Bahrain Defence Force, Royal Medical Services (BDF-RMS), Bahrain; b Salmaniya Medical Complex (SMC), Bahrain

**Keywords:** Peutz-Jeghers syndrome, Sertoli cell tumor, Gynecomastia, Case report

## Abstract

Sertoli cell tumors (SCTs) are a rare occurrence, accounting for 0.4 %–1.5 % of all testicular masses. They are characterized as classic, large cell calcifying, or sclerosing Sertoli cell tumors depending on their clinical characteristics. These tumors can be associated with multiple genetic disorders, one of which is Peutz-Jeghers syndrome (PJS). PJS is a result of the mutation of STK11/LKB1 gene, manifesting as oral hyperpigmentation, benign colonic polyps, gastrointestinal and non-gastrointestinal tumors. Here we describe a case of intratubular large cell hyalinizing Sertoli cell tumor, a particularly rare variant of SCTs, with the detailed steps in diagnosis and management.

## 1 Introduction

Peutz-Jeghers Syndrome (PJS) is an autosomal dominant disease presenting with mucocutaneous pigmentation and hamartomatous polyps in the gastrointestinal tract.^1^ It is also associated with multiple malignancies, including gastrointestinal, testicular and ovarian tumors. With regards to testicular tumors, patients with PJS carry a cumulative risk of 9 %, with a strong predisposition for Sertoli cell tumors (SCT).^2^ These patients typically present with gynecomastia and sexual precocity. We describe a case in which a presentation of gynecomastia led to the diagnosis of SCT and previously undiagnosed PJS.

## 2 Case presentation

An 8-year-old Arab boy presented with a 1-year history of bilateral gynecomastia and no other previously known comorbidities or surgical history. The breast swelling was progressive and painful in nature, and it affected the left side more than the right. On examination, bilateral breast enlargement and tenderness were noted. However, no distinct breast lump or mass was felt. There was also a lack of facial, axillary and pubic hair growth appropriate for his age. On genitalia examination, testes were bilaterally enlarged (about 10ml) with no darkening of scrotal skin. However, no distinct lesion or mass was palpable and no hydrocele or hernia was detectable on examination. Phallus size was appropriate for current age. There were no palpable lymph nodes in the axillary, neck, supraclavicular and inguinal regions. On inspection of oral mucosa, mucocutaneous oral freckling and pigmentation were identified, and similar lesions were also found on the patient's father.

## 3 Investigations

**Fig. 1:**
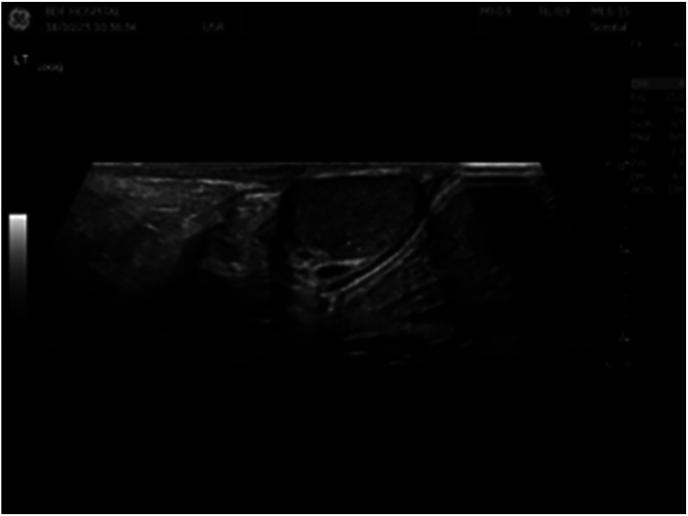
Microlithiasis on ultrasonography of testes.

**Fig. 2:**
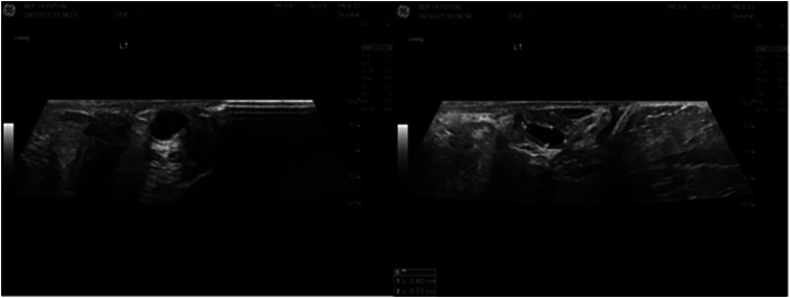
Benign epididymal cyst in left testis.

A hormonal panel was done and yielded results within normal range. Breast ultrasonography was done and showed bilateral prominent breast tissue, left more than right, keeping with gynecomastia. Both axillae were unremarkable. Testicular enlargement was confirmed on ultrasound examination with a testicular volume of 6 ml (lambert formula). In addition, microlithiasis was also noted (Fig. 1) with a left benign epididymal cyst (Fig. 2). However, no definite testicular mass was visualized at the time. In view of his clinical presentation and prior investigations, genetic testing was performed which identified a heterozygous variant in the STK11 gene, confirming the diagnosis of Peutz-Jegher disease.

**Fig. 3:**
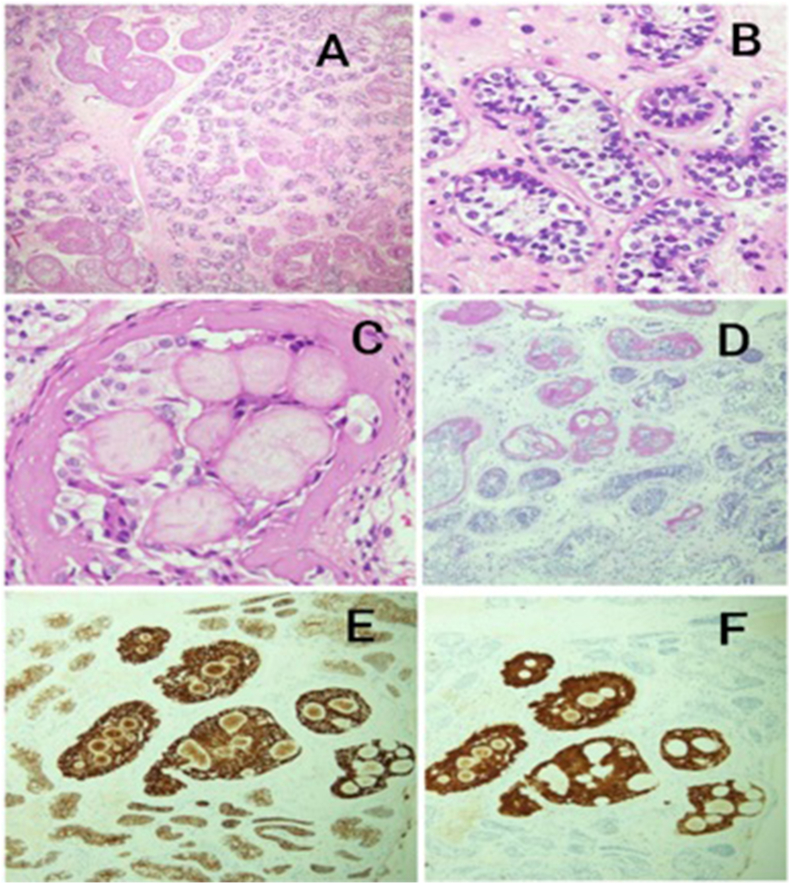
A: low power view shows scattered markedly expanded seminiferous tubules with eosinophilic appearance, B: Immature seminiferous tubules lined by infantile spermatogonia and Sertoli cells, C: eosinophilic basement like material with intraluminal round eosinophilic deposits, D: PAS special stain highlights basement like material, E: IHC; Inhibin, F: IHC; Calretinin.

Given the association between PJS and testicular tumors, ultrasound findings, and clinical picture of increased hormonal activity, there was suspicion of a secreting testicular lesion. To rule this out, the patient underwent right testicular lesion biopsy. Histopathological assessment of biopsy showed immature seminiferous tubule lined by infantile spermatogonia and Sertoli cells. In addition, there was an increased group of cells between the seminiferous tubules having round nuclei and light eosinophilic cytoplasm with eosinophilic cytoplasmic granules (Fig. 3). On immunohistochemistry, the cells were positive for calretinin, Beta-catenin, strong positive for inhibin A, weak positive SF1, intermediate positive for CD99. These cells were also surrounded by eosinophilic basement like material with occasional intraluminal round eosinophilic deposits, that were positive for PAS and PAS-D but negative for Congo red, with thick interstitial blood vessels. No features of granuloma, active inflammation, calcification or malignancy were seen. These histopathological findings are consistent with large cell hyalinizing Sertoli cell tumor (LHCSCT).

## 4 Management

The patient was referred to a pediatric endocrinologist for initiation of aromatase inhibitors and genetic tests for his siblings were offered. He is scheduled to have an ultrasound after 6 months to survey for the development of a future testicular mass.

**Fig. 4:**
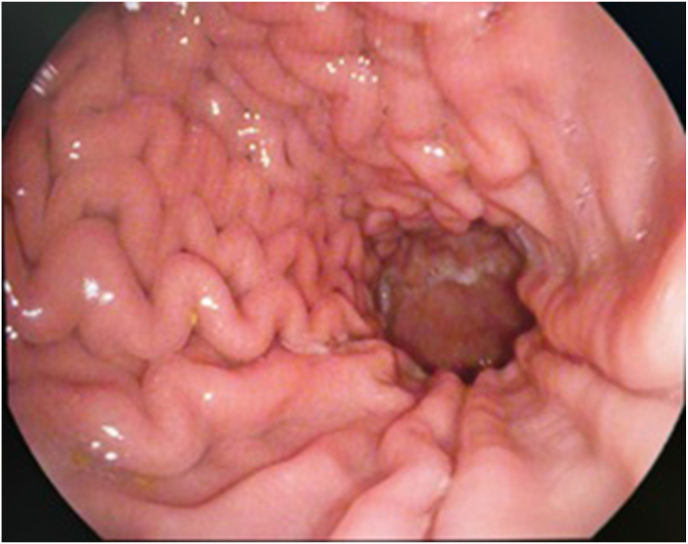
Stomach polyp on upper GI endoscopy.

Additionally, gastrointestinal surveillance was done by a pediatric gastroenterologist through upper GI endoscopy and colonoscopy. The upper GI endoscopy revealed a normal esophagus, 2 small polyps in the stomach, 3 sessile polyps in the first part of the duodenum, and a normal second part (Fig. 4). The colonoscopy showed 2 small sigmoid polyps, otherwise unremarkable. Biopsies were taken and revealed the following: Active chronic gastritis, mild acute inflammation of duodenal mucosa, and acute chronic inflammation of colonic mucosa. No signs of dysplasia or malignancy were appreciated in histological assessment.

## 5 Discussion

Sertoli cells are generally classified into three entities: Sclerosing, large cell calcifying and not otherwise specified (NOS). Large cell hyalinizing Sertoli cell tumors (LCHSCTs) were historically recognized as part of Sertoli cell NOS tumors. However, in 2016 the WHO classifies LCHSCTs as a separate entity with its own morphology and distinct features, almost exclusively found in boys with Peutz-Jegher syndrome (PJS) with a unique STK11 gene mutation.^3^ The relationship between PJS and LCHSCTs is well-established; increased expression of aromatase due to STK11 gene mutation results in the manifestation of prepubertal bilateral gynecomastia and Sertoli cell neoplasia. This is further supported by aromatase staining demonstrated on Sertoli cell histopathology.^4^

SCTs usually occur over a wide range of age groups, from 15 to 80 years, most commonly affecting patients aged 45.^3^ The presenting feature is typically a slowly enlarging testicular mass that may also be painful in some cases.^5^ Looking at LCHSCTs, a previous study showed 7 of 8 patients with bilateral gynecomastia as the presenting symptom. Furthermore, all patients had bilateral enlarged testes on examination but no distinct mass. Hormonal panels demonstrated elevated estradiol (>8pg/ml).^6^

Under microscopy, features of LCHSCTs include patchy expanded tubules filled with Sertoli cells and globular eosinophilic basement membrane deposits connecting with thick peritubular basement membranes.^7^ Sertoli cells themselves demonstrate pale to eosinophilic appearances with vacuolated cytoplasm. The mixed appearance of basement membrane deposits alongside the expanded tubules gives LCHSCTs their hyalinized appearance. In immunohistochemistry, Sertoli cell tumors typically show positivity for inhibin-a and calretinin, helping to differentiate it from other testicular tumors such as seminoma.^8^ CD99 expression is another characteristic finding in Sertoli cell tumors used to aid diagnosis.

In this case, the patient presented with progressive painful breasts bilaterally, whereas most SCT cases initially present with testicular mass. Alongside clinical and radiological findings, pathological evaluation is crucial in the diagnosis of Sertoli cell tumors. Microscopic features such as intratubular Sertoli cells with an eosinophilic appearance and vacuolated cytoplasm alongside positivity for immunohistochemical markers such as inhibin, beta-catenin, calretinin and CD99 are vital for definitive differentiation between Sertoli cell tumors and other testicular tumors.

Regarding management, an aromatase inhibitor was the choice of treatment for this patient given the link between the disease and aromatase expression. The aromatase enzyme typically converts androgen into estrogen, leading to symptoms of hormonal overexpression such as gynecomastia and precocious puberty.^9^ A study in which young patients with PJS were started on aromatase inhibitor therapy showed a decrease in gynecomastia, a normalization of growth velocity and a delay in premature skeletal maturation without requiring orchidectomy.^10^

## 6 Conclusion

Sertoli cell tumors continue to be a rare entity with limited literature and discussion. This is especially true for more scarce subtypes such as large cell hyalinizing SCTs. For these cases, clinical assessment is a key feature in helping identify early features of the disease, with laboratory investigations and radiology used to support the diagnosis. However, definitive diagnosis requires pathological assessment with immunohistochemistry. The primary treatment involves hormonal therapy along with further workup for underlying syndromes and management as needed.

## CRediT authorship contribution statement

**Rashed Almusalam:** Writing – original draft, Visualization, Methodology, Investigation, Formal analysis, Data curation, Conceptualization. **Shaikha Janahi:** Writing – original draft, Methodology, Formal analysis, Data curation. **Mohammed Basem:** Writing – original draft, Visualization, Methodology, Formal analysis. **Hasan Isa:** Visualization, Investigation. **Abdulrahman Alshafei:** Writing – review & editing, Validation, Supervision, Project administration, Conceptualization.

## Authors' contributions

All authors have contributed equally to this research project. Moreover, they have all reviewed the final draft of this case report.

## Consent for publication

This case report involved a pediatric patient, consent to publish was granted by their father.

## Funding

The authors of this case report have not received any funding for this research project.

## Conflict of interests

The authors declare that they have no known competing financial interests or personal relationships that could have appeared to influence the work reported in this paper.
